# Top-Down Modulation on Perceptual Decision with Balanced Inhibition through Feedforward and Feedback Inhibitory Neurons

**DOI:** 10.1371/journal.pone.0062379

**Published:** 2013-04-23

**Authors:** Cheng-Te Wang, Chung-Ting Lee, Xiao-Jing Wang, Chung-Chuan Lo

**Affiliations:** 1 Institute of Systems Neuroscience, National Tsing Hua University, Hsinchu, Taiwan; 2 Center for Neural Science, New York University, New York, New York, United States of America; 3 Department of Neurobiology and Kavli Institute for Neuroscience, Yale University School of Medicine, New Haven, Connecticut, United States of America; 4 Department of Life Science, National Tsing Hua University, Hsinchu, Taiwan; Georgia State University, United States of America

## Abstract

Recent physiological studies have shown that neurons in various regions of the central nervous systems continuously receive noisy excitatory and inhibitory synaptic inputs in a balanced and covaried fashion. While this balanced synaptic input (BSI) is typically described in terms of maintaining the stability of neural circuits, a number of experimental and theoretical studies have suggested that BSI plays a proactive role in brain functions such as top-down modulation for executive control. Two issues have remained unclear in this picture. First, given the noisy nature of neuronal activities in neural circuits, how do the modulatory effects change if the top-down control implements BSI with different ratios between inhibition and excitation? Second, how is a top-down BSI realized via only excitatory long-range projections in the neocortex? To address the first issue, we systematically tested how the inhibition/excitation ratio affects the accuracy and reaction times of a spiking neural circuit model of perceptual decision. We defined an energy function to characterize the network dynamics, and found that different ratios modulate the energy function of the circuit differently and form two distinct functional modes. To address the second issue, we tested BSI with long-distance projection to inhibitory neurons that are either feedforward or feedback, depending on whether these inhibitory neurons do or do not receive inputs from local excitatory cells, respectively. We found that BSI occurs in both cases. Furthermore, when relying on feedback inhibitory neurons, through the recurrent interactions inside the circuit, BSI dynamically and automatically speeds up the decision by gradually reducing its inhibitory component in the course of a trial when a decision process takes too long.

## Introduction

Neurons in the central nervous systems are continuously bombarded by noisy excitatory and inhibitory synaptic inputs with roughly balanced and covaried intensity. This balanced synaptic input (BSI) has been observed in various regions including the frontal cortex [1], [2], primary visual cortex [3], developing primary auditory cortex [4], somatosensory barrel cortex [5] and spinal cord [6]. Experiments showed that the balance in excitation and inhibition is crucial in stabilizing the neural circuit when receiving excitatory input [5], [7]. It has also been suggested that imbalance in the ratio of excitation to inhibition in the brain may contribute to certain psychiatric disorders [8], [9]. At the single neuron level, BSI has also been demonstrated to provide a source of background noise that increases overall conductance and response variability of neurons [10]–[12]. Furthermore, recent computational and experimental studies have shown that BSI modulates the response property of neurons [13]–[15] and hence provides a plausible mechanism for gain modulation at the neuronal level [16]–[21]. A key insight is that with the ability of changing the gain of single neurons, BSI may play more proactive roles than previously thought in exerting top-down control over neural circuit functions.

Indeed, a number of studies have shown that BSI shapes the tuning curve of sensory neurons [3], [4], [20]. Furthermore, some of us have previously demonstrated that in a neural circuit model of perceptual decision [22]–[25], by applying BSI with different strengths we could dynamically adjust performance of the decision process in terms of trading between speed and accuracy [26]. The speed-accuracy tradeoff (SAT) is a salient feature of decision making [27]–[30] and is commonly described as the result of adjusting a decision threshold in a drift diffusion model (DDM) [28], [30]–[33]. A number of studies suggested that the decision threshold adjustment may be implemented in the cortico-basal ganglia circuit [22], [28], [34], [35]. Our model provided a different (but not mutually exclusive) neuronal mechanism of SAT and predicted that the ramping rate of neural integrators for information accumulation is higher with speed emphasis, which is consistent with a recent electrophysiological experiment with behaving monkeys [36].

Although BSI provides an appealing idea for the top-down and dynamic control of the neural circuit functions, two important issues remain to be investigated: 1. In the highly noisy and plastic neural circuits, the ratio between the inhibition and excitation in BSI may not be able to maintain at an ideal level from trial to trial. Therefore, we ask how the ratio affects the functions of BSI. 2. The top-down control is presumably provided by long-range projections from remote cortical regions such as prefrontal cortex [37]–[40] which is known to be crucial for executive control. Considering that cortical output neurons are typically excitatory pyramidal neurons which can provide the excitatory component of BSI, we ask how the inhibitory component of BSI can be produced by the long-range excitatory projection.

To address the issues, we systematically investigated the behavior (reaction times and task performance) and the dynamics of the neural circuit under the influence of BSI with different ratios using the neural circuit model of perceptual decision [22]–[25]. We further investigated a novel BSI configuration in which the long-range projection from a remote cortical brain region is excitatory only and the balanced inhibition is established within the decision circuit. Specifically, we assumed that the long-range excitation projects onto both pyramidal neurons and GABAergic interneurons in the feedback decision circuit in light of recent findings of long-range projection from the prefrontal cortex onto the GABAergic neurons in other brain regions [41]–[43].

We found that BSI produces rich effects on the decision process. There exist two operational modes of BSI and each corresponds to different ranges of BSI ratio. In one region, increasing the top-down control (BSI strength) speeds up the decision whereas in the other region, increasing the top-down control improves the accuracy. We also found that BSI can be established internally within the feedback decision circuit by the GABAergic interneurons. Furthermore, this internally produced BSI gradually shifts to the excitatory side as the decision progresses. This change of balance provides an internal signal that speeds up the decision when it takes too long.

## Methods

### The Perceptual Decision Task: Random-dot Motion Discrimination

**Figure 1:**
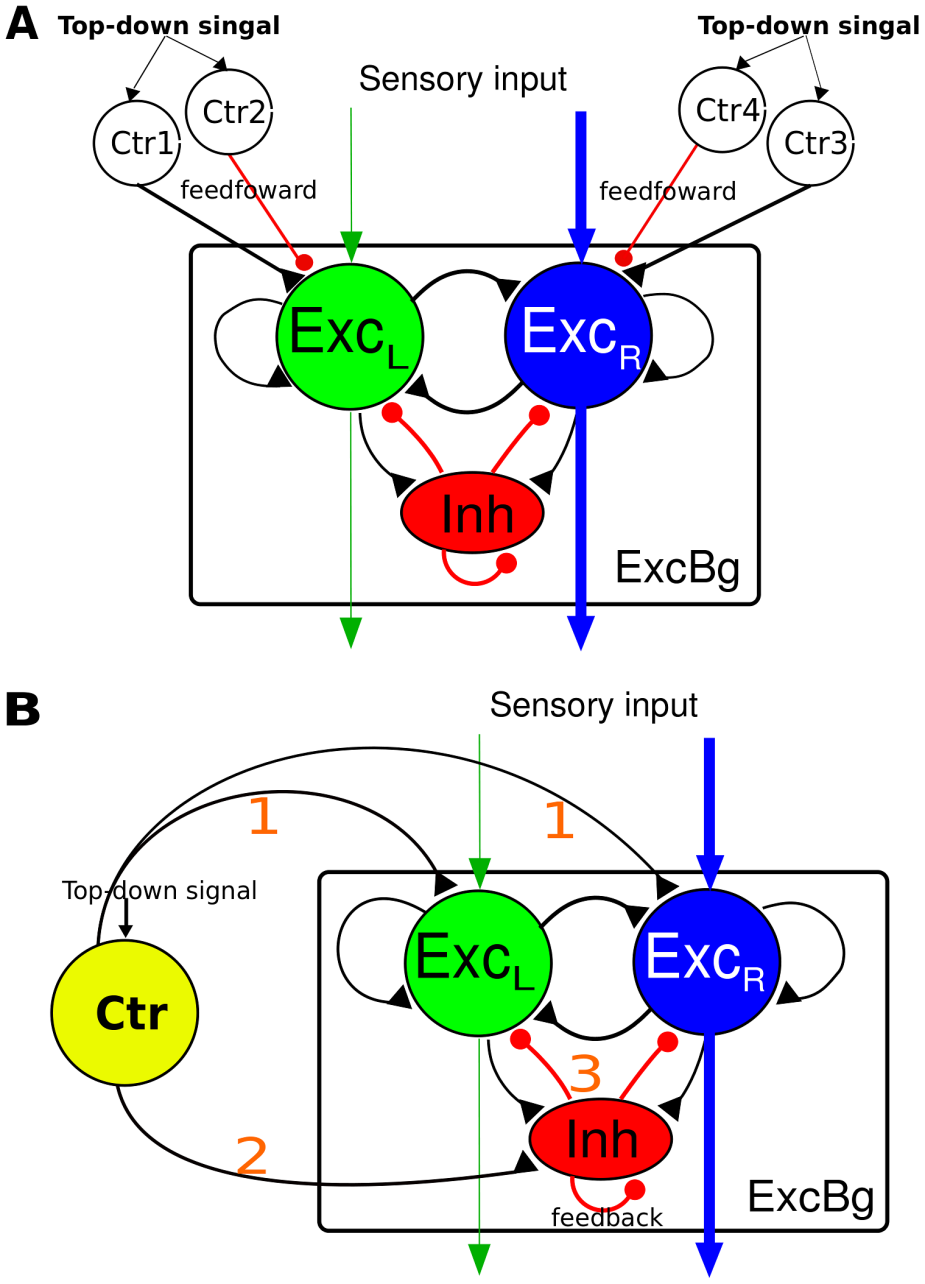
Schematics of a cortical neural circuit model of perceptual decision with different configurations of balanced synaptic input (BSI). The basic model circuit consists of two strongly-recurrent populations of excitatory neurons (Exc_L_ and Exc_R_) serving as the decision neurons and a population of inhibitory interneurons (Inh) which produces mutual inhibition between the two excitatory populations. There is an excitatory background neural population (ExcBg) that is not selective to task-relevant stimuli and maintains a baseline activity. The two decision populations receive sensory inputs and compete against each other by ramping up its activity and suppressing the other. A decision is made when the population firing rate of a decision population crosses a preset threshold first. A. In first configuration, BSI_ff_, the balanced excitatory and inhibitory inputs to Exc_L_ and Exc_R_ are generated externally by two pairs of excitatory and inhibitory neuronal populations (ctr1-ctr4) in a feedforward manner. B. In the second configuration, BSI_fb_, the balance between excitation and inhibition is generated internally through the feedback inhibitory neurons. The circuit receives a long range excitatory projection from a top-down control module (Ctr) to all neurons in the circuit. The balance between the excitation and inhibition is determined internally by the input strength of Ctr -> Exc (pathway 1) and the increased input strength (comparing to the condition without the Ctr input) of Inh -> Exc (pathway 3) which is driven by Ctr neurons through the pathway 2.

In the present study we investigated the effects of BSI on perceptual decision by performing model simulations of a visual direction-discrimination task using the random-dot motion paradigm [44]. In the task, a subject is shown a display of randomly moving dots. A small portion (called coherence level or stimulus motion strength) of the dots move coherently toward one of the two possible directions, e.g. right or left. The subject is required to determine the direction of the coherent motion. The subject has to indicate the direction by a saccadic eye movement as soon as a decision is reached. In our model, two inputs (presumably from middle temporal area (MT) as previously reported [45]) representing the amount of rightward and leftward random-dot motion directions are fed into neurons in two competing neural populations (Exc_R_ and Exc_L_) in the decision circuit model, respectively (Figure 1). The mean spike rate μ of each input depends on the motion strength of the stimulus linearly and follows the equations: μ = μ_0_ + µ_A_ × c’ for the direction of the coherent motion and μ = μ_0_ - µ_B_ × c’ for the opposite direction. μ_0_ ( =  40 Hz) is the baseline input for the purely random motion, c’ (between 0% and 100%) is the coherence level that characterizes the stimulus motion strength and µ_A_ ( =  120 Hz) and µ_B_ ( =  40 Hz) are factors of proportionality. The differences between the values of µ_A_ and µ_B_ is to capture the observation in which the population average of the slope of MT neuron response function was found to be roughly 3.5 times higher in the preferred direction than in the non-preferred direction [45]. Given the fact that µ_A_ and µ_B_ for individual MT neurons follow broad distributions [45], our assumption of µ_A_ = 3 µ_B_ is not substantially different from the observation. In the present study, we used six levels of motion strength: 0%, 3.2%, 6.4%, 12.8%, 25.6% and 51.2%. The decision time is defined as the time interval between the start of the sensory input and the time when the population firing rate of either of the populations Exc_L_ and Exc_R_ reaches a preset threshold (30 Hz). The reaction time is defined as the decision time plus a 250 ms non-decision time which represents the neural processing time not related to the decision. A correct trial is defined as the trial in which the excitatory population (Exc_L_ or Exc_R_) which receives the stronger input (μ_0_ + µ_A_ × c’) reaches the decision threshold first.

### Neural Circuit Model

The computational model used in the present study is based on a previously described cortical circuit model [22]–[25] that is capable of working memory and perceptual decision [22]–[24], [29], [46]–[51]. Briefly, the network model consists of four interconnected neural populations Exc_R_, Exc_L_, Inh and ExcBg (Figure 1). Each of Exc_R_ and Exc_L_ contains excitatory neurons and receives inputs that represent the random-dot moving toward right and left, respectively. The populations compete against each other through the population I which consists of inhibitory interneurons. The non-selective background population ExcBg contains 1100 excitatory neurons mimicking neurons that are selective for directions other than the two forced-choice alternatives or to other stimuli that are irrelevant to the present study. See Table 1 for the number of neurons, background noise input and connectivity of the circuit model. In the model, ExcBg neurons do not receive stimulus input and maintain a baseline activity (several Hertz). The circuit model exhibits winner-take-all competition: only one of the excitatory populations (Exc_R_ or Exc_L_) can win the competition by ramping up its activity until it crosses the decision threshold, whereas the other population is eventually suppressed. This behavior resembles neuronal activity observed in the lateral intraparietal area and frontal eye fields in monkeys when performing the random-dot task or other tasks such as visual search [36], [44], [52].

**Table 1:** The parameters for each neural population.

Name	Number ofneurons	Background noise(rate / conductance in nS)	Target Population(target receptor: conductance in nS)
ExcBg	1120	2400 / 2.1	ExcBg (A: 0.05), ExcBg (N: 0.165)
			Exc_L_ (A: 0.0429), Exc_R_ (A: 0.0429)
			Exc_L_ (N: 0.142), Exc_R_ (N: 0.142)
			Inh (A: 0.04), Inh (N: 0.13)
Exc_R_	240	2400 / 2.1	ExcBg (A: 0.05), ExcBg (N: 0.165)
			Exc_L_ (A: 0.0429), Exc_L_ (N: 0.142)
			Exc_R_ (A: 0.09), Exc_R_ (N: 0.297)
			Inh (A: 0.04), Inh (N: 0.13)
Exc_L_	240	2400 / 2.1	ExcBg (A: 0.05), ExcBg (N: 0.165)
			Exc_R_ (A: 0.0429) Exc_R_ (N: 0.142)
			Exc_L_ (A: 0.09), Exc_L_ (N: 0.297)
			Inh (A: 0.04), Inh (N: 0.13)
Inh	400	2400 / 1.62	ExcBg (G: 1.398), Exc_L_ (G: 1.398)
			ExcR (G: 1.398), Inh (G:1.075)
Ctr1 ( 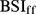 )	500	x / 2.1	Exc_L_ (A: 0.1)
Ctr2 ( 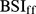 )	500	x / 2.1	Exc_L_ (G: x)
Ctr3 ( 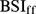 )	500	x / 2.1	Exc_R_ (A: 0.1)
Ctrl4 ( 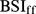 )	500	x / 2.1	Exc_R_ (G: x)
Ctr ( 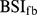 )	240	x / 2.1	ExcBg (A: 1), Exc_L_ (A: 1),
			Exc_L_ (A: 1) , Inh (A:x)

In the fourth column, “A” indicates AMPA receptors, “N” indicates NMDA receptors and “G” indicates GABA_A_ receptors. “x” indicates variable values that were used to set the strength and ratio of BSI.

### Single Neuron and Synapse Models

Each neuron in the circuit model is simulated using the leaky integrate-and-fire model. The membrane potential *V(t)* for each neuron obeys the following equation:
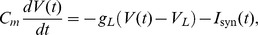
where *C_m_* is the membrane capacitance, *g_L_* is the leak conductance, *V_L_* is the resting potential and *I*_syn_ is the total synaptic current. When the membrane potential *V*(*t*) of each neuron reaches a threshold *V*_threshold_ = −50 mV, a spike is emitted and *V(t)* is set to the reset potential *V*_reset_ = −55 mV for a refractory period *T_r_* = 2 ms. For inhibitory neurons, we used the following parameters: *C_m_* = 0.2 nF, *g_L_* = 20 nS and *V_L_* = −70 mV. For excitatory neurons, we used *C_m_* = 0.5 nF, *g_L_* = 25 nS and *V_L_* = −70 mV.

The synaptic current *I*_syn_ *(t)* includes inputs from visual stimulus, other neurons in the circuit (recurrent connections), background noise and the top-down control input:
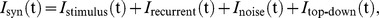
where the background noise is applied to all neuronal populations and visual stimulus are only applied to the populations Exc*_R_* and Exc*_L_*. In the case of BSI_ff_, only the two decision populations Exc*_R_* and Exc*_L_* receive the top-down control input whereas in the case of BSI_fb_, all populations receive the top-down control input. We modeled three types of receptors for synapses: AMPA, NMDA and GABA_A_. They are described by:



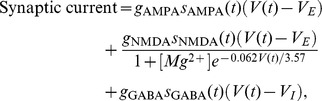
where V*_E_* ( = 0) and V*_I_* ( = −70 mV) are the reversal potentials, [Mg^2+^] ( = 1.0 mM) is the extracellular magnesium concentration, *g* is the synaptic efficacy and s is the gating variable. Subscripts in *g* and *s* denote the receptor type. The gating variables of the three receptors obey



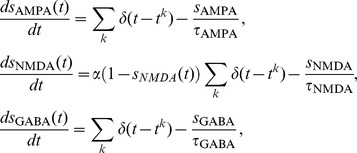
where the decay constants 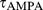 =  2 ms, 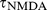 = 100 ms and 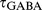 =  5 ms. α = 0.63. δ*(t−t^k^)* is the delta function and *t^k^* is the time of the *k*th presynaptic spike.

All synaptic connections between neural populations and within a neural population (recurrent connections) are all-to-all, i.e. every neuron in the source population makes synaptic connections to every neuron in the target populations. Each neuron in the network receives external spike inputs (spike rate = 2400 Hz) through AMPA mediated receptor with Poisson statistics serving as the background noise. See Table 1 for the detailed setting.

### Balanced Synaptic Input (BSI)

Neurons in the populations Exc_R_ and Exc_L_ receive BSI, in which the excitatory and inhibitory components are applied through AMPA and GABA_A_ mediated receptors, respectively. Given a ratio between the strength of the excitatory and inhibitory components of BSI, the induced depolarizing and hyperpolarizing currents can cancel each other (balanced) at a specific membrane potential, *V_B_*. This can be formularized as:
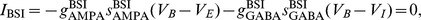
where the superscript BSI denotes the balanced synaptic input. Assuming that BSI provides a steady input with a mean firing rate *r*, we can easily demonstrate that the steady input results in a mean gating variable *s* = *τr*. Let spike rate of the excitatory and inhibitory components of BSI be *r_e_* and *r_i_*, respectively. We have



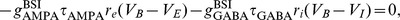
which leads to



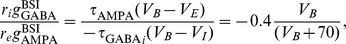
where τ_GABA_ = 5 ms and τ_AMPA_ = 2 ms. The left side of the equation, 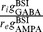, represents the ratio between the strength of inhibitory and excitatory components of BSI and is hence defined as BSI ratio. The equation shows that there is no single value of the BSI ratio that cancels the hyperpolarizing and depolarizing currents for all levels of the membrane potential. Rather, for a given membrane potential V*_B_*, we can find a specific BSI ratio which produces balanced hyperpolarizing and depolarizing currents. The effect of BSI is that it drives the membrane potential toward V*_B_* which acts as a reverse potential. When the membrane potential is higher than V*_B_*, BSI produces a hyperpolarized current. If the membrane potential drops below V*_B_*, the BSI current becomes depolarized.

### BSI through Feedforward and Feedback Inhibitory Neurons

In the present work, we investigated BSI in two different configurations. In the first configuration the balanced inhibition is established through feedforward inhibitory neurons (BSI_ff_) (Figure 1A) and in the second configuration the balanced inhibition is established through the feedback inhibitory neurons (BSI_fb_) (Figure 1B). In BSI_ff_, excitatory and inhibitory inputs are projected to the excitatory decision population Exc_L_ and Exc_R_ in a feedforward manner. Each of the excitatory and inhibitory components is provided by an independent neural population. Therefore, *r_e_* and *r_i_*, can be controlled independently by driving the corresponding neural populations (Ctr_1_-Ctr_4_) with a top-down signal implemented by Poisson spike inputs. We define two important parameters, BSI_ff_ ratio and strength:
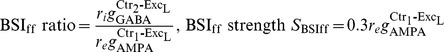



Since all parameters are symmetric between the left side (Exc_L_, Ctr_1_ and Ctr_2_) and the right side (Exc_R_, Ctr_3_ and Ctr_4_) of the neural circuit, here we only define the BSI_ff_ ratio and strength using the parameters in one side. We note that BSI_ff_ ratio is only defined for the condition of non-zero BSI_ff_ strength (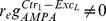). In the model *r_e_* and *r_i_* are represented by the firing rate of each neurons in the excitatory and inhibitory control modules, respectively. The purpose of multiplying 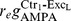 by 0.3 was to bring the value of the maximum working BSI_ff_ strength to about 1 as 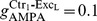 nS and we found that the maximum working value of *r_e_* is in the range of 30–40 Hz. In the simulations, changes of the BSI_ff_ strength were done by changing the firing frequencies (*r_e_* and *r_i_*) of both AMPA and GABA inputs by the same percentage (thus leaving the BSI_ff_ ratio unchanged), whereas changes of BSI_ff_ ratio were done by changing the value of GABA conductance (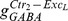). Theoretically changing the firing frequency and changing the input conductance result in the same effect as the input strength equals the multiplication of the two factors. The reason why we chose this way of varying BSI_ff_ strength and ratio was to be consistent with the way we varied the strength and ratio of BSI_fb_ (see below).

In BSI_fb_, we tested a more biologically realistic setting in which the top-down influence is originated from a control module (Ctr) via a long-range cortical projection, which is excitatory only. We assumed that the projection provides non-specific excitatory input to all neural populations in the circuit model. The excitatory component of BSI_fb_ is provided by the direct input from the top-down control module (Ctr) to Exc_L_ and Exc_R_ while the inhibitory component is provided by the increased firing rate *dr*_inh_ (due to the input from the top-down module) of the inhibitory interneurons in Inh. However, *dr*_inh_ is influenced by the neural interaction in the circuit and is not directly controllable by the top-down control module. What is controllable is the strength of the input (including the spike rate and the synaptic strength) from the control module to Exc_L_ (and Exc_R_) and Inh. Therefore, we define a “preset” BSI_fb_ ratio as the ratio between the two inputs:
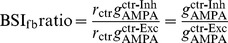




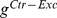
 here represents both 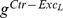 and 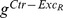 because they are of the same value in the current model. *r*_Ctr_ is the firing rate of each neuron in the control module, which can be controlled by the background input to the module.

We found that the maximum working BSI_fb_ strength is *r*_Ctr_∼14 Hz. Given that the control module in BSI_fb_ has 240 neurons with a synaptic strength 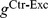 of 1 nS and the control module in BSI_ff_ has 500 neurons with a synaptic strength 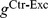 of 0.1 nS, *r*_Ctr_ = 14 Hz in BSI_fb_ matches *r_e_* = 67.2 Hz in the strength in BSI_ff_, or *S*_BSIff_∼2.0. Accordingly, we define BSI_fb_ strength as
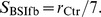



This makes *S*_BSIfb_ roughly match the strength of *S*_BSIff_ in values. We note that the normalization factors (0.3 for BSI_ff_ and 1/7 for BSI_fb_) are specific to our modeling setting and the purpose is only to make the comparison between BSI_ff_ and BSI_fb_ easy. In the simulations we varied the strength of BSI_fb_ by changing the firing frequency (*r*_Ctr_) of the control module and varied the ratio of BSI_fb_ by changing the conductance (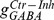) of the input from the control module to the inhibitory neurons.

### Estimation of the Energy Function

We constructed a one-dimensional energy function to shed insights into the circuit dynamics affected by BSI. Briefly, we considered the difference in the firing rates of the two selective neural populations, 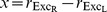. In each trial, the change of *x* over each time window of 20 ms was assessed as an estimate of the velocity of the network dynamics, 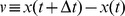, where 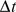 = 20 ms. We choose 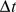 = 20 ms as the firing rates were calculated using a time window of 20 ms in our simulator. Note that *v(x)* is strongly influenced by the noise in the circuit. To filter out the noise, we calculated *v(x)* for each time steps ( = 0.1 ms) of the simulation for 1000 trials and obtained a large pool of data. We then calculated average *v(x)* for each small range of *x* from the pooled data to obtain a smooth *v(x)* in which the noise are averaged-out. The energy function, or the potential, *U(x)* was obtained based on 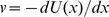 [24], [53], by integrating *v* over *x*: 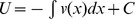, where *C* is a constant of integration which can be chosen arbitrarily without affecting the analysis of the system dynamics. To avoid the edge effect which distorts the values of *v(x)*, we discarded the data points for which 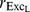 < 3 Hz when *x* > 0 and 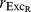 < 3 Hz when *x* < 0. The edge effect arises from the fact that the firing rates of Exc*_R_* and Exc*_L_* cannot go below zero. If the firing rate of either Exc_R_ or Exc_L_ is very close to zero, it is likely to increase in the next time steps due to the noise perturbation, thereby introducing a bias in *v*(*x*). For example, if *x* is positive (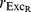
> 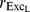) while 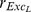 ≈ 0 at a given time *t*, *x* tends to decrease at *t*+Δ*t* due to increasing 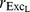
_._ This produces a negative *v*(*t*) even though 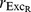 may also increase.

### Spike Synchronization

In the study we investigated the effect of BSI on spike dynamics by measuring the spike synchrony in the decision population (Exc_R_ or Exc_L_). We adapted the algorithm published in Quiroga et al. 2002 [54]. Briefly, the algorithm counts the number of events in which spikes from two neurons fall within a preset time window τ ( = 5 ms in the present study) and then normalizes the event count by the spike numbers of the two neurons. The procedure results in a non-negative index of spike synchronization with the maximum value of 1 representing a pair of fully synchronized spike trains.

## Results

**Figure 2:**
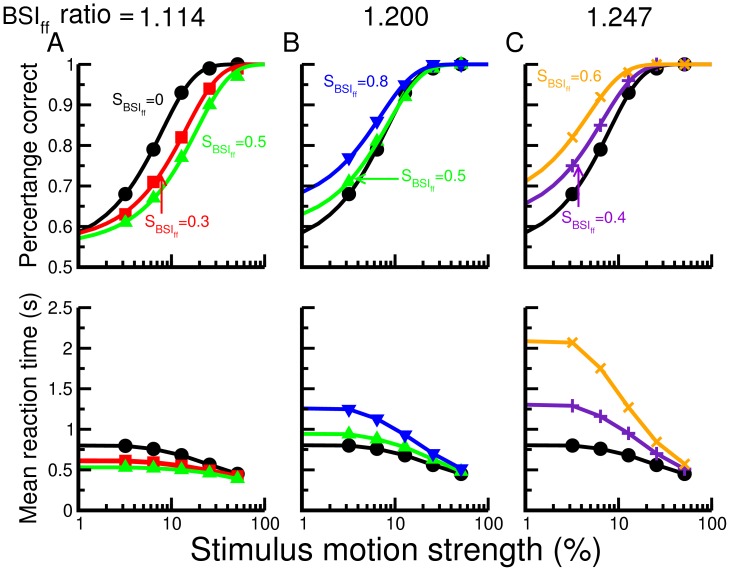
The behavioral outcome of the decision circuit is dependent on the BSI_ff_ strength and ratio A. Performance (top), defined as the portion of trials with correct decisions, and mean reaction time (bottom) as functions of the task difficulty (characterized by the stimulus motion strength) for different BSI_ff_ strength (black = 0, red = 0.3, green = 0.5). BSI_ff_ ratio = 1.11. B. Same as in A with ratio = 1.20 (BSI_ff_ strength: black = 0, green = 0.5, blue = 0.8). C. Same as in A with ratio = 1.25 (BSI_ff_ strength: black = 0, purple = 0.4, orange = 0.6). Depending on the ratio, the performance and mean reaction time change differently with increasing BSI_ff_ strength. With a higher ratio, stronger BSI_ff_ increases the speed of decision (shorter mean reaction time) while decreases the performance. With a lower ratio, we found an opposite trend in which a stronger BSI_ff_ increases the performance while reduces the speed of decision. The curves in the top panels in A-C are plotted only for the visualization purpose only. The curves were obtained by fitting to the data using the function 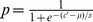 where c’ is the stimulus motion strength, 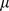 and s are fitting parameters.

We first tested how different ratios between inhibition and excitation (BSI ratio) affect the behavior of the decision circuit. To this end, we performed the test with the condition of BSI_ff_ in which the excitatory and inhibitory components of BSI are all provided and controlled independently by external sources (Figure 1A). We found that when the BSI_ff_ ratio is low (more excitation), stronger BSI_ff_ reduces the performance (percentage of correct decisions) but increases the speed of decision (Figure 2A). This is because the extra excitation triggers faster ramping activity which reduces the time for the system to integrate the sensory input. If we decreases the BSI_ff_ ratio (increasing the strength of the inhibitory component), the BSI-induced changes in performance and mean reaction time become smaller and smaller until at a certain ratio the effect reverts and BSI starts to produce better performance and slower reaction time (Figure 2 B–C). The result suggests that BSI is able to exhibit two different modulatory effects simply by changing the ratio between its excitatory and inhibitory components.

**Figure 3:**
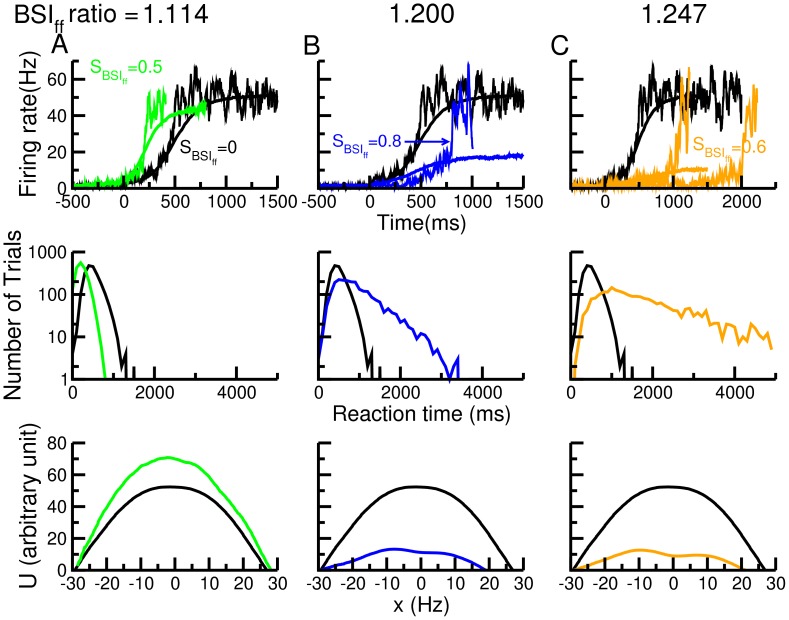
BSI_ff_ modulates the neuronal activity and the dynamics of the decision circuit differently between different BSI ratios. A. At a lower BSI_ff_ ratio ( = 1.11), the ramping rate of the population firing activity of the winning decision population increases with BSI_ff_ strength (top). Thick curves show trial-averaged population firing rate and thin curves are samples of population firing rate from single trials. The effect of changing ramping activity is reflected in the shape of the reaction time distribution (middle). The faster ramping rate caused by increasing BSI_ff_ strength results from the steeper energy landscape (bottom). *x* represents the difference between the population firing rates of Exc_L_ and Exc_R_. The stimulus motion strength is 3.2% for all conditions. BSI_ff_ strength = 0 (black) and 0.5 (green) B. Same as in A with a higher BSI_ff_ ratio ( = 1.20). With more inhibition, BSI_ff_ causes an opposite effect: the ramping rate decreases with increasing BSI_ff_ strength. The slowing down in the ramping activity is due to the shallower energy landscape around the peak. When the BSI_ff_ strength is strong enough, a crater is created at the center of the peak which significantly slows down the speed of decision (falling into one of the two basins). BSI_ff_ strength = 0 (black) and 0.8 (blue) C. The trend becomes more significant with a stronger inhibitory component in BSI_ff_ (ratio = 1.25). BSI_ff_ strength = 0 (black) and 0.6 (orange).

We next investigated how the neural activity and its dynamics are changed by BSI_ff_. With more excitation in BSI (ratio = 1.11), stronger BSI leads to a faster ramping activity (Figure 3A top) for the wining neural population, which is consistent with the trend of shorter mean reaction time. When we increased the inhibitory component, BSI starts to slow down the ramping activity (Figure 3B &C, top). Interestingly, the slowing down is characterized by not just one, but two trends: 1) a smaller ramping slope and 2) a delayed onset time of the ramping activity. We looked at the behavior change from a different aspect by plotting the reaction time distribution (Figure 3 A-C, middle). The result showed that when the mean reaction time is increased by a strong BSI_ff_, the effect is not simply due to a shift of the distribution, but also due to the production of a long tail. The long tail indicates that while we still see many fast decisions as in no BSI_ff_ conditions, we also observe some trials with extremely long reaction time.

The trends of delayed onset of the ramping activity and the slower ramping activity observed in the simulations can be explained by considering the energy function of the neural circuit (Figure 3A–C, bottom). With a low BSI ratio, the stronger excitatory component in BSI makes neurons in the decision neural populations (Exc_L_ and Exc_R_) more excitable. The effect is that once a population wins the competition, it tends to accelerate its ramping activity due to the stronger recurrent excitation. The dynamical change is reflected in the energy landscape (Figure 3A bottom) which shows a steeper slope. On the other hand, with a high BSI ratio, the stronger inhibitory component reduces the excitability of the neurons and hence weakens the competition between Exc_L_ and Exc_R_. The weakened competition slows down the accumulation of neuronal activity of the wining population and also produces a relatively stable state in the beginning of a trial when the firing rates of the two populations are comparable. These dynamical changes are reflected in the energy landscape by the appearance of a crater on top of the peak and a gradual slope. When a trial starts, the system tends to stay in the crater, or the energy well, for a period of time until the noise in the system pushes the system out of the energy well. Once the system leaves the energy well, it falls into either side of the peak and makes a decision. The period which the system stays in the energy well corresponds to the delayed onset time of the ramping activity. The speed of ramping is determined by the slope of the energy landscape outside the crater (Figure 3C).

**Figure 4:**
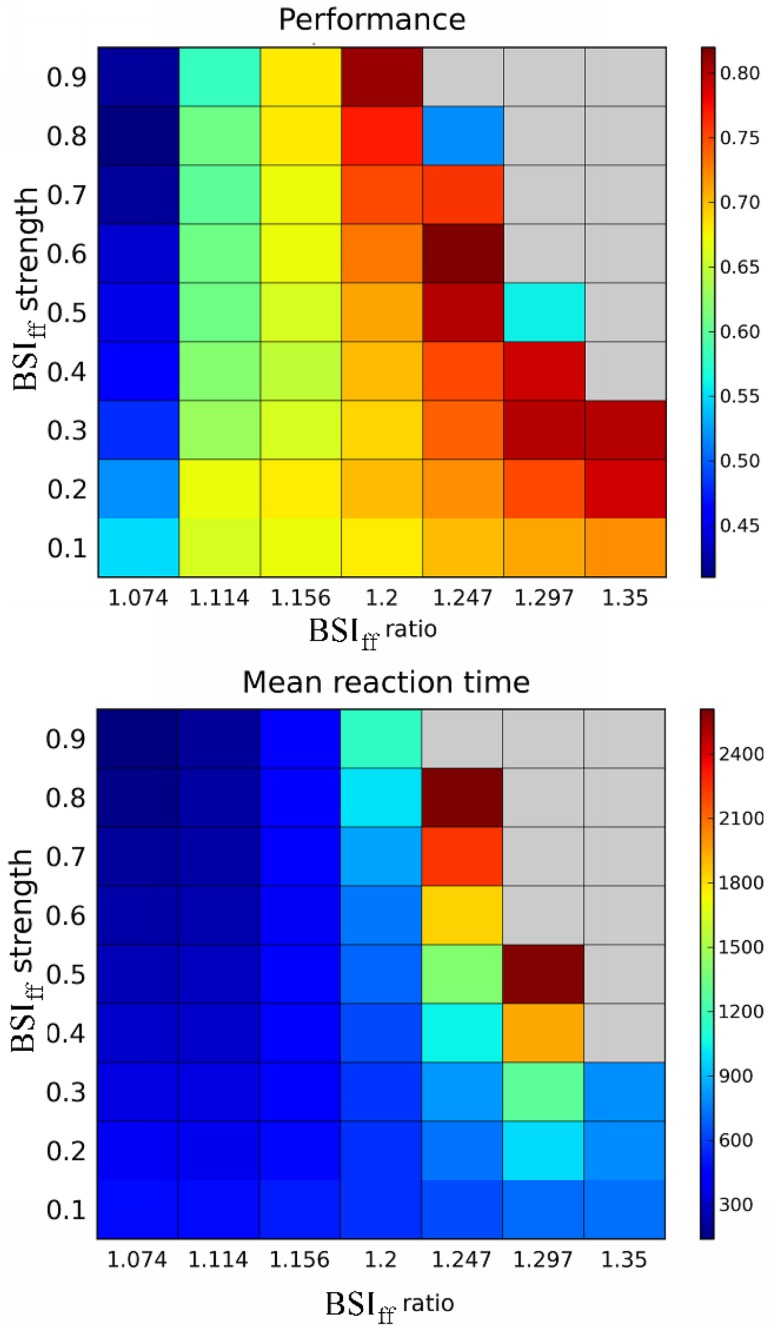
Effects of BSI_ff_ on A, task performance (portion correct) and B, mean reaction time across different values of BSI_ff_ ratio and strength for the stimulus motion strength c’ = 3.2%. There is a critical value of the ratio ( = 1.156). Above the value, the performance and the mean reaction time increase with increasing BSI_ff_ strength. In contrast, the performance and reaction time decrease with increasing BSI_ff_ strength when the ratio is below the critical value. The gray regions indicate that under the given strength and ratio, the system could not reach a decision in more than 5% of the trials, hence we excluded them from the analysis.

We systematically tested the behavioral effect of BSI_ff_ and plot the performance and mean reaction time as functions of BSI_ff_ ratio and strength (Figure 4). We observed a critical value of BSI_ff_ ratio (∼1.156). Below this critical value, increasing BSI_ff_ strength reduces the performance and the mean reaction time whereas above the critical value, increasing BSI_ff_ strength improves the performance but also prolongs the mean reaction time. At the critical value, changing BSI_ff_ strength does not significantly affect the performance and the mean reaction time. As a summary, the effects of BSI_ff_ on the decision behavior can be characterized by two operational modes: a “speed-emphasis” mode that corresponds to the BSI_ff_ ratios below the critical value and an “accuracy-emphasis” mode for the BSI_ff_ ratios above the critical value. Therefore, in a noisy neural circuit environment, if the system cannot maintain a constant ratio of BSI_ff_, it can still exhibit the same modulatory effect on the perceptual decision as long as the ratio stays in the same side of the critical value. We note that the gray regions in Figure 4 indicate that under the given strength and ratio, the system could not reach a decision in more than 5% of the trials, hence we excluded them from the analysis. These non-decision trials were characterized by extremely slow ramping activity due to strong inhibition in BSI_ff_ and therefore have decision times longer than the cut-off time.

We have demonstrated the basic properties and effects of BSI which is implemented by feedforward excitation and inhibition. Next, we consider a more realistic BSI configuration in which BSI is applied through a long range intra-cortical projection. The idea is that if BSI acts as a top-down control that modulates the perceptual decision, the modulation is likely originated from higher brain centers such as prefrontal cortex [37]–[40]. Considering that the intra-cortical projections are typically excitatory only, one plausible way to produce balanced excitation and inhibition is to have these long-range excitations project to both excitatory neurons and inhibitory interneurons in the decision circuit. The projection excites the interneurons hence increases their inhibitory inputs to the excitatory decision neurons. The increased inhibitory inputs act as the inhibitory component of BSI (Figure 1B). Because the inhibitory component is generated by the inhibitory interneurons which are part of the feedback local decision circuit, we define this type of BSI as BSI_fb_. The strength of BSI_fb_ is represented by the firing rate of the long-range excitatory input (from the control module Ctr) while the BSI_fb_ ratio can be tuned by changing the synaptic weight of the Ctr-Inh projection. This is because a stronger synaptic weight induces stronger activity in Inh neurons, hence increases the BSI ratio.

**Figure 5:**
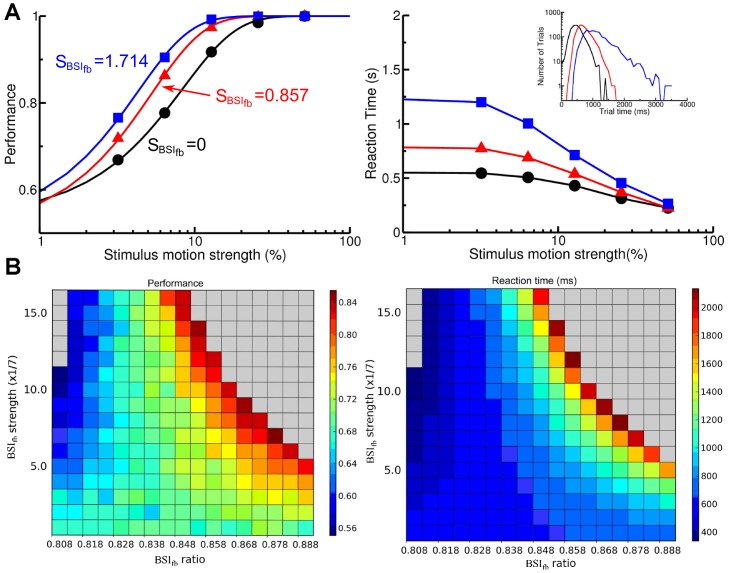
BSI_fb_ modulates behavior of perceptual decision in a way similar to that of BSI_ff_. A. Performance (left) and mean reaction time (right) are shown for three different BSI_fb_ strengths (black: 0, red: 0.857, blue: 1.714) with the BSI_fb_ ratio of 0.848. At this ratio, a stronger BSI_fb_ increases the performance and mean reaction time. The curves in the left panel are plotted for visualization purpose only and were obtained by curve fittings using the same function as described in Figure 2 caption. B. A summary of the effect of BSI_fb_ strength and ratio on the performance (left) and the mean reaction time (right) with the stimulus motion strength of 3.2%. Similar to that of BSI_ff_, with a lower ratio (more excitation), increasing BSI_fb_ strength speeds up the decision process whereas with a higher ratio (more inhibition), increasing BSI_fb_ strength improves the performance. As in Figure 4, the gray regions indicate that under the given strength and ratio, the system could not reach a decision in more than 5% of the trials.

We tested the behavior performance of BSI_fb_ by varying its strength and ratio (Figure 5) and found that BSI_fb_ influenced the behavior of the decision circuit in a way similar to BSI_ff_. With a low BSI ratio, increasing BSI_fb_ strength shortens the mean reaction time but reduces the performance whereas with a high BSI ratio, increasing BSI_fb_ strength improves the performance but prolongs the mean reaction time. The accuracy-emphasis and speed-emphasis modes are separated by the critical ratio = 0.828. We note that the gray regions in Figure 5B indicate the conditions in which the system could not reach a decision in more than 5% of the trials. The non-decision trials in the gray regions in the upper right corners correspond to the case in which no neural population reaches the decision threshold until end of the trial as in the gray regions in Figure 4. In contrast, the non-decision trials in the gray regions in the upper left corners correspond to the case in which both neural populations ramp up and reach the threshold together due to too much excitation in BSI_fb_.

**Figure 6:**
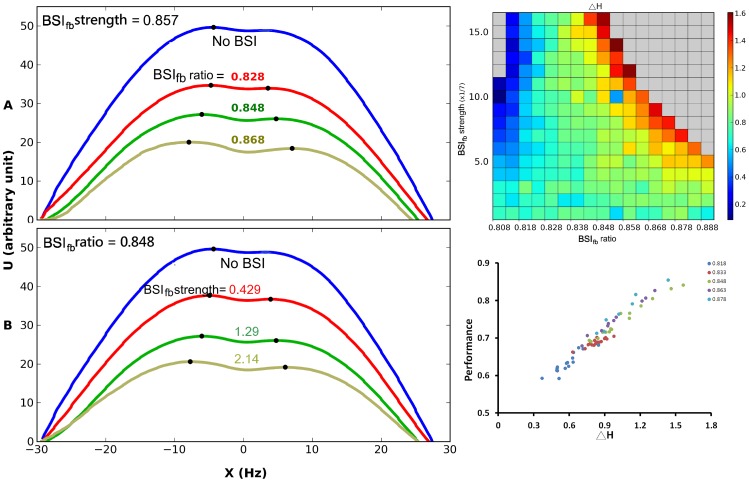
BSI_fb_ alters the dynamics of the circuit by changing its energy landscape in a way similar to BSI_ff_. A. (Top) Energy landscape for different BSI_fb_ ratios. The BSI strength is set to be 0.857. If we increase the BSI_fb_ ratio, the slope of the landscape becomes shallower while the crater on top of the hill becomes bigger and deeper. (Bottom) Energy landscape for different BSI strengths. The BSI_fb_ ratio is set to be 0.848. A similar trend can be observed if we increased the BSI strength. B. The differences between the heights of the two walls of the crater (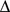
H) as a function of BSI_fb_ ratio and strength. C. Performance as a function of 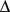
H plotted for several BSI_fb_ ratios and strengths. Each color indicates data obtained from one BSI_fb_ ratio and each dot of a given color represents a specific BSI_fb_ strength for the corresponding BSI_fb_ ratio. The data from different BSI_fb_ ratios form a linear relationship with 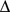
H with overlapping distributions, which indicate that the performance is mainly determined by the size of the crater. Regardless a specific combination of BSI_fb_ ratio and strength, as long as they give rise to the same 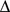
H, the performance of decision is the same. The stimulus motion strength c’ = 3.2% in all panels.

We further investigated how BSI_fb_ changes the dynamics of the circuit. We plotted the energy functions for different BSI_fb_ strengths and ratios (Figure 6A). We found that, similar to BSI_ff_, the energy function is altered by BSI_fb_. With a medium BSI_fb_ strength (0.857) and ratio (0.848), higher ratios or stronger BSI_fb_ strengths produce a shallower energy landscape with a wider and deeper crater on the hill. We define 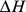
as the differences between heights of the left wall and right wall of the crater. We found that 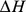
varies with the BSI_fb_ ratio and strength in a way very similar to what the performance does (Figure 6B, comparing to Figure 5B left panel). In the low ratio region (BSI_fb_ ratio < 0.828), increasing BSI_fb_ strength reduces 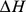
while in the high ratio region, increasing BSI_fb_ strength enlarges 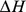. From the dynamical system point of view, the height of the wall of an energy well determines the probability of a noisy system overcoming the barrier and jumping out of the energy well. Therefore, the difference between the heights of the two walls of the crater determines the probability of the system escapes from one sides versus the other side. Indeed, we found that 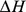 ultimately determines the performance of the system (Figure 6C). Regardless of the BSI_fb_ ratio and strength, as long as the neural circuit experiences the same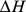, they performance similarly.

**Figure 7:**
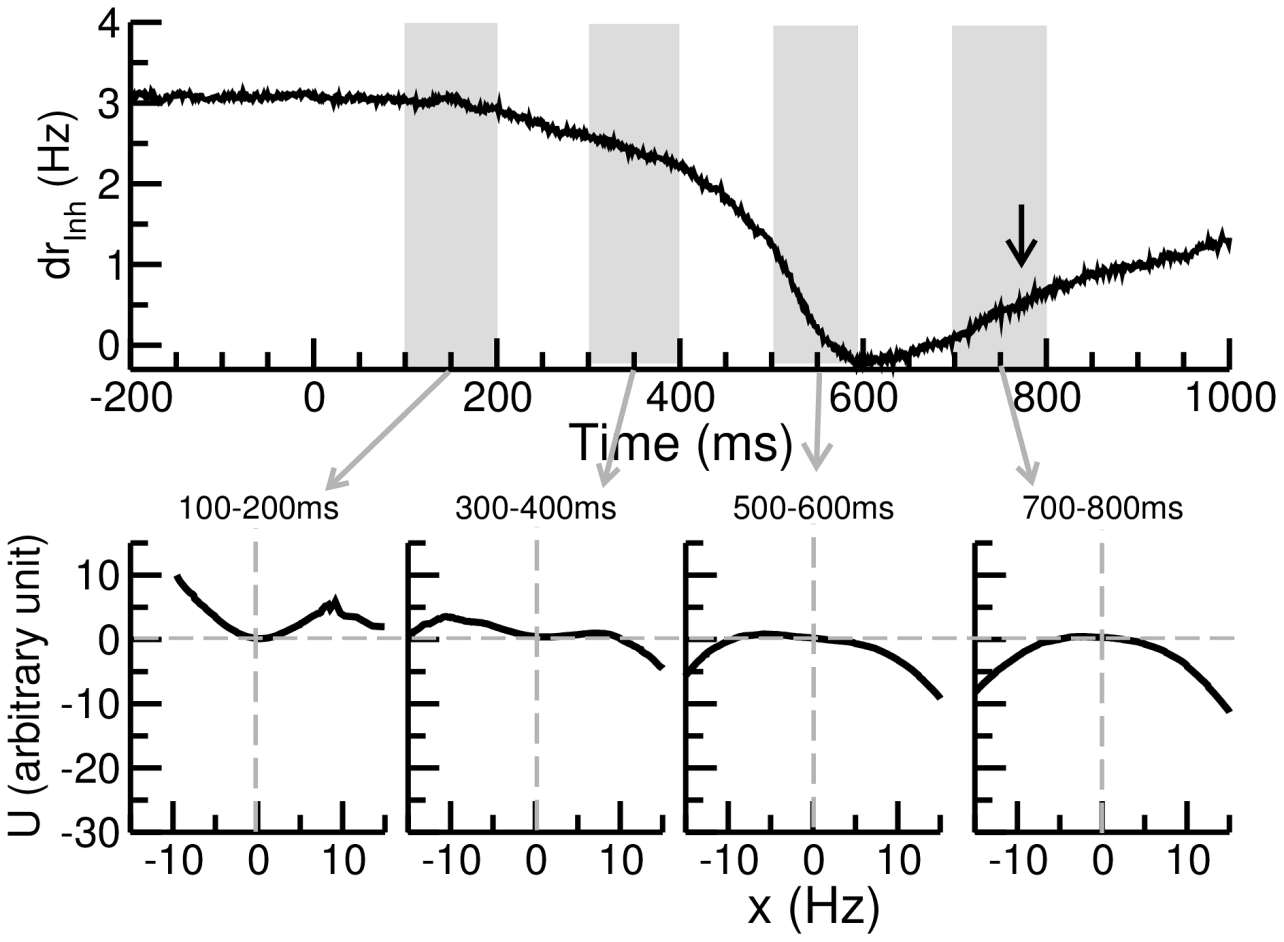
BSI_fb_ produces time varying inhibitory component and the energy landscape in the course of a trial. A. The time progression of the inhibitory component (*dr*_inh_) of BSI_fb_. With a given BSI_fb_ ratio ( = 0.848) and strength ( = 0.857), the balance between the excitation and inhibition of BSI_fb_ changes with time after the stimulus onset. The inhibitory component reduces gradually, indicating a trend of shifting toward excitation in BSI_fb_ during the decision process. The black arrow indicates the mean reaction time. The motion strength c’ = 3.2%. B. The instantaneous energy landscapes are shown for four different periods: 100–200 ms, 300–500 ms, 500–600 ms and 700–800 ms. The result indicates that the center crater of the energy landscape gradually disappears during the course of a trial. This change provides an internal mechanism to speed up the decision when it takes too long.

Due to the strong and nonlinear interactions between neurons in the circuit, the actual inhibitory component *dr*_inh_ (the difference of Inh firing rate between the conditions with and without BSI_fb_) may change during the course of a trial even when the top-down input from Ctr neurons remains constant. A time varying *dr*_inh_ indicates a changing BSI_fb_ ratio, or a changing dynamics of the neural circuit. To accurately estimate the instantaneous *dr*_inh_ in the course of a trial, we calculated the trial-average *dr*_inh_ from 2000 trials. We selected a moderate BSI_fb_ condition (BSI_fb_ ratio = 0.848, strength = 0.857), and calculated trial-averaged *r*_inh_ as a function of time for the two conditions (with a moderate BSI_fb_ and without BSI_fb_). The difference in *r*_inh_ between the two conditions gives rise to the inhibitory component *dr*_inh_. We found that the instantaneous *dr*_inh_ starts to drop around 200ms after the stimulus onset, reaches the minimum at 600 ms and rises again slowly (Figure 7A).

To evaluate the impact of the varying strength of the inhibitory component of BSI_fb_ on the network dynamics, we calculated the instantaneous energy function at three representative periods: 100–200 ms, 400–500 ms and 700–800 ms after the stimulus onset. We observed an intriguing phenomenon which was in consistence with the trend of decreasing *dr*_inh_ as shown in Figure 7A: the top region of the energy landscape gradually transforms from an earlier “trapping state” (with a crater) to a latter “force-decision” state (without the crater) (Figure 7B). In the trapping state the system stays in the crater (*r*_ExcL_∼*r*_ExcR_) for a prolonged time period due to the local stability in the crater until the system is pushed away by the noise. The trapping state often results in extremely long reaction times. Therefore, the disappearance of the crater in the later part of a trial in the BSI_fb_ condition forces the system to move down the hill and prevents long reaction times, implying that the system has an intrinsic mechanism to speed up the decision when it takes too long.

**Figure 8:**
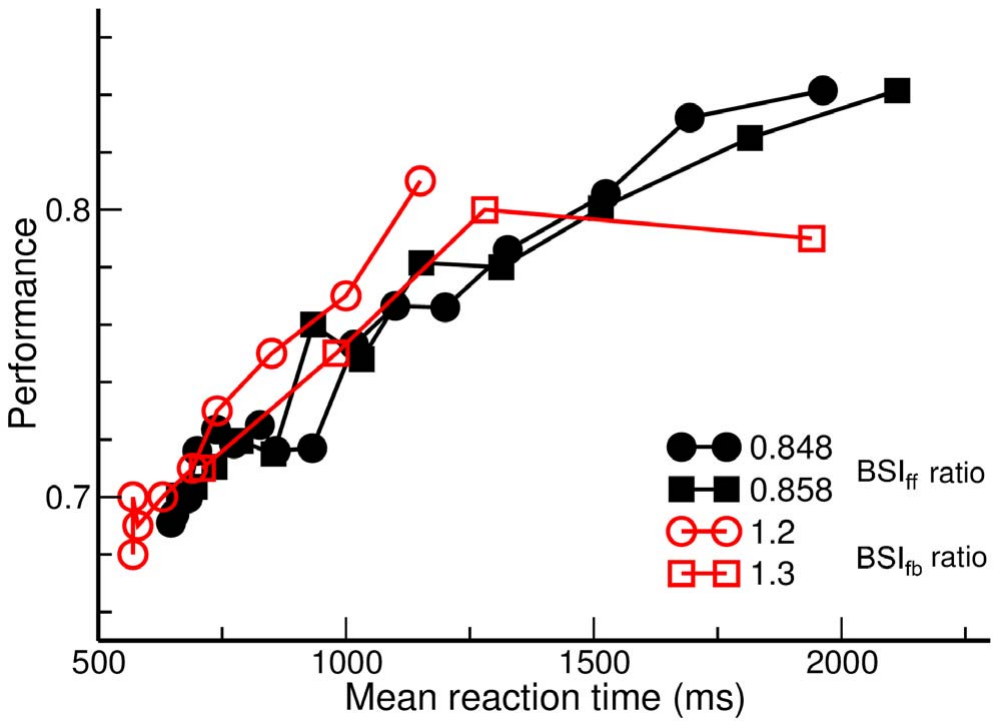
BSI_fb_ produces a wider range of speed-accuracy tradeoff. We plot performance versus mean reaction time with motion strength = 3.2% for the BSI_fb_ (black curves) and BSI_ff_ (red curves) conditions. In the working range of BSI_fb_ (preset ratio = 0.848−0.858), the system exhibits trading between speed and accuracy in wide ranges of mean reaction time and performance while for BSI_ff_ (ratio = 1.2−1.3), speed-accuracy trade-off works in a narrower range. With BSI_ff_, when the mean reaction time exceeds 1300 ms, the performance is not improved anymore.

We further investigated how the time-varying energy landscape affects the behavior performance. To this end, we tested how the performance improves differently with increasing BSI strength in the BSI_ff_ and BSI_fb_ conditions (Figure 8). We choose the c’ = 3.2% condition because both BSI_fb_ and BSI_ff_ exhibit the greatest performance improvement when the task is difficult. We found that BSI_fb_ produces a wide range of speed-accuracy tradeoff and the performance reaches ∼85% with a mean reaction time of ∼2.0s whereas BSI_ff_ produces a smaller performance improvement which tops at 80% with a mean reaction time of ∼1.5s. The reason of a smaller performance improvement is that BSI_ff_ produces a fix-shaped energy landscape which has a deep crater on the top of the energy landscape when BSI_ff_ is strong. A deep crater increases the risk that the system being trapped in the non-decision state (the center crater) until the end of the trial which has a 5000 ms cut-off time after the onset of the motion stimulus. Therefore, when BSI_ff_ is strong, the reaction time increases rapidly and the increasing probability of non-decision trial reduces the performance. On the other hand, there is no such concern for BSI_fb_ as the crater gradually disappears as the trial progresses.

**Figure 9:**
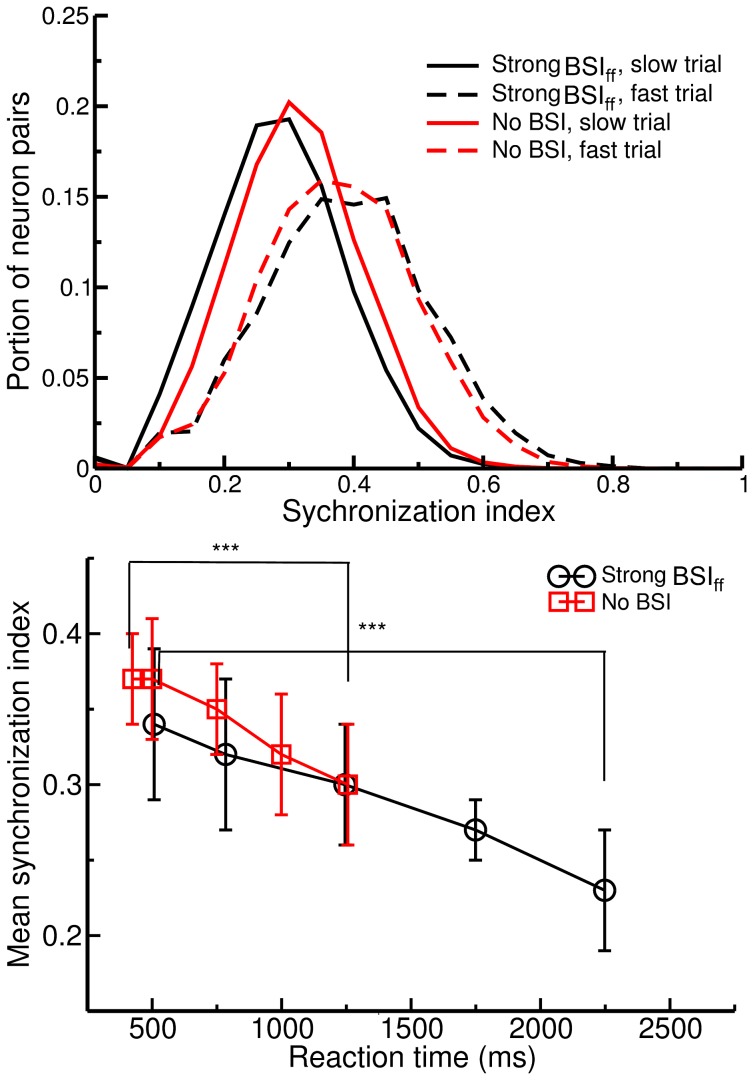
Degree of spike synchronization between neurons significantly correlates with the reaction time, but not with the balanced synaptic input. A. Distributions of the synchronization index between each pair of neurons in the wining decision population (Exc_R_ or Exc_L_) for different trial conditions. Each distribution represents one selected trial. We selected trials with fast (reaction time ∼600 ms) and slow (reaction time ∼1300 ms) responses from both BSI conditions (BSI_ff_ strength = 0.5 and no BSI). We can find clear difference between the distributions of slow and fast trials, but not between strong BSI_ff_ and no BSI trials with similar reaction times. B. Mean synchronization index reduces significantly with reaction time for both strong BSI_ff_ and no BSI conditions. Each point represents the mean synchronization index averaged over 10 trials with similar reaction times. Error bars indicate the standard deviation and “***”s denote p<0.0005 in Student’s t test.

We have shown how BSI modulates the decision process from dynamical system point of view by demonstrating the variations in the energy landscape. One may ask whether the changes at the system level reflect a more fundamental change in the spike dynamics. To this end, we measured the spike synchronization (see Method) between each pair of neurons in the winning decision population (Exc_R_ or Exc_L_) for various conditions including strong BSI_ff_ (strength = 0.5), no BSI, fast trials and slow trials (Figure 9). We found that, while the mean spike synchronization reduces with the reaction time, there is no significant difference in the spike synchrony between the strong BSI_ff_ and no BSI trials with similar reaction times. Therefore, the effect of BSI mainly lies in the firing rate at circuitry levels rather than in the individual spike levels, at least for the proposed model.

## Discussion

In the present study, we have demonstrated that by varying the strength of the input and the ratio between the excitatory and inhibitory components of BSI_ff_, we can switch the neural circuit between two operational modes (speed emphasis or accuracy emphasis). The result suggests that BSI_ff_ is an efficient and versatile mechanism for the top-down modulation of the decision process. We further investigated internally generated BSI, in which the decision neural circuit only receives an excitatory long-rang projection as the top-down control. We showed that if the excitatory projection innervates all neurons including excitatory neurons and inhibitory interneurons in the decision circuit, a balance between the excitation and inhibition can still be achieved. In fact, this BSI_fb_ exhibits time-varying dynamics which prevents the circuit from being trapped in a deadlock state and thus improves the tradeoff between speed and accuracy.

Our finding of two operational modes for BSI is interesting because it suggested that the same mechanism can accommodate two distinct behavioral effects. In the “speed-emphasis” mode, which corresponds to lower BSI ratios (stronger excitation), increasing BSI strength speeds up the decision. In contrast, in the “accuracy-emphasis” mode, which corresponds to stronger inhibition, increasing BSI strength improves the accuracy. The result implies that in some perceptual decision tasks, we may observe that the activity of the brain region which exerts the top-down control increases in response to time pressure (speed instruction) [55], whereas in other tasks we may observe the top-down modulation increasing its activity in response to performance pressure (accuracy instruction) [56]. Our model suggests that the seemly contradictory observations could result from the same mechanisms in two different operational modes. Moreover, while the top-down control from the remote brain region may represent the overall level of attention, whether the top-down influence should be used to increase the speed or the accuracy is determined locally in the decision circuit through learning induced synaptic plasticity. For example, assuming the system is initially in the speed-emphasis mode (lower BSI ratios) when the task requires a better accuracy. A strong top-down control is likely to reduce the accuracy and the number of rewards which results in no synaptic weight change or slight depression in ctr-Exc synapses (due to the low dopamine level). If occasionally in some trials the inhibitory neurons become strongly activated. The strong activation increases BSI ratio which improves the accuracy and results in more rewards. As a consequence, the ctr-inh synapses are facilitated due the co-activation of the control and inhibitory neurons and the circuit gradually shifts toward higher BSI ratio region, or the accuracy-emphasis mode. It is interesting to implement such a learning mechanism in future studies and see how the model quantitatively reproduces some of the behavioral observations.

We note that the differential responses of the top-down control observed between studies could also result from other factors such as the task designs, types of decision and actual functions of the brain regions being studied. For example, a brain region could exert a top-down control to modulate the information accumulation (as proposed here), or to change the decision threshold [22], [28], [34], [35]. Further studies with carefully designed decision tasks are needed in order to test our model predictions.

To address the issue that the long-range projection of the top-down control is likely to be excitatory only, we proposed BSI_fb_ in which the inhibitory interneurons in the decision circuit participate in producing the inhibitory component of BSI. However, one may argue that the issue can also be addressed by BSI_ff_ if the feedforward inhibitory neurons in local to the decision circuit. However, this solution requires a dedicated inhibitory neural population which is local but does not receive any input from other neurons in the local circuit and it is not clear whether such type of neurons exist in the cerebral cortex. In contrast, our BSI_fb_ proposal does not need a new neural population but only utilizes the existing inhibitory interneurons that already participate in the decision process.

Another interesting property we discovered in the present study is how the central crater in the energy landscape determines the performance of the system. Although the performance is affected by both BSI_fb_ strength and ratio at the circuitry level, it all boils down to the shape of the central crater of the energy landscape from the perspective of dynamical system. We found that regardless of the BSI_fb_ strength and ratio, as long as 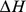
(the differences between the heights of the two walls of the central crater) is the same, the system exhibits the same performance (Figure 6C).

Our result of time-varying BSI_fb_ ratio within a trial (Figure 7) suggests a natural way to prevent the neural circuit from being trapped in the deadlock state. To improve the performance, one needs to increase 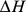, which produces a side effect of a wider and deeper crater. In the case of BSI_ff_ in which the BSI ratio remains constant throughout the whole trial, the wider and deeper crater significantly reduces the probability of the system jumping out of the crater, hence increases the expected time for the system to reach a decision. When the crater is big enough, the reaction times become extremely long and the performance starts to drop because the system fails to jump out of the crater and reaches a decision before the timeout (5000 ms) in some trials. This indicates the limitation in the performance improvement by BSI_ff_. On the other hand, in the case of BSI_fb_, the BSI ratio starts to decrease after the onset of the trial and the crater gradually disappears, hence the system is less likely to be trapped in the deadlock state. Therefore, the decreasing BSI ratio can be viewed as a mechanism generated internally to speed up the decision when the decision process becomes too long. Comparing to the decision made under BSI_ff_, BSI_fb_ can further improve the performance without losing too much in the reaction time (Figure 8).

In the model we only considered AMPA mediated currents for the excitatory component in BSI_ff_ and BSI_fb_ due to the consideration of simplicity. Ideally both AMPA and NMDA should be taken into account because the excitatory (glutamatergic) input is often simultaneously mediated by both receptor types. However, NMDA receptors have the same reverse potential with that of AMPA, therefore both types of receptors have a similar effect in BSI. The change in the strength of excitatory component of BSI due to the omission of NMDA mediated current can be easily compensated by increasing the strength of AMPA conductance. Although NMDA receptors are endowed with voltage dependent conductance, during the course of a trial the membrane potential of an excitatory neuron mainly varies in the range between the reset potential (−55 mV) and the threshold potential (−50 mV), resulting in small (only ∼7%) changes in the NMDA conductance. Therefore the voltage dependent NMDA receptors do not significantly alter the property of BSI and it is safe to omit NMDA when we analyze the effects of BSI.

In summary, our result provides three novel perspectives regarding how top-down control in a form of balanced synaptic input modulates perceptual decision: 1) by changing the ratio between the excitation and inhibition in the balanced synaptic input, one can switch the neural circuit between the accuracy-emphasis mode and the speed-emphasis mode, suggesting that the two different types (or modes) of top-down modulation can be realized by the same mechanism, 2) the balanced synaptic input can be produced internally with the participation of inhibitory interneurons in the decision circuit when the top-down control input is excitatory only. Therefore, there is no need for recruiting additional inhibitory neurons that are dedicated for balancing the top-down excitatory input, and 3) the BSI_fb_ exhibits time-varying ratio between the inhibition and excitation, which provides an internal signal that speeds up the decision and improves the ability of the neural circuit to trade speed for accuracy.
